# Evolving stochastic context--free grammars for RNA secondary structure prediction

**DOI:** 10.1186/1471-2105-13-78

**Published:** 2012-05-04

**Authors:** James WJ Anderson, Paula Tataru, Joe Staines, Jotun Hein, Rune Lyngsø

**Affiliations:** 1 Department of Statistics, University of Oxford, 1 South Parks Road, UK; 2 Bioinformatics Research Centre, Aarhus University, C. F. Møllers Allé 8, Denmark; 3 Department of Computer Science, University College London, Gower Street, UK

## Abstract

**Background:**

Stochastic Context–Free Grammars (SCFGs) were applied successfully to RNA secondary structure prediction in the early 90s, and used in combination with comparative methods in the late 90s. The set of SCFGs potentially useful for RNA secondary structure prediction is very large, but a few intuitively designed grammars have remained dominant. In this paper we investigate two automatic search techniques for effective grammars – exhaustive search for very compact grammars and an evolutionary algorithm to find larger grammars. We also examine whether grammar ambiguity is as problematic to structure prediction as has been previously suggested.

**Results:**

These search techniques were applied to predict RNA secondary structure on a maximal data set and revealed new and interesting grammars, though none are dramatically better than classic grammars. In general, results showed that many grammars with quite different structure could have very similar predictive ability. Many ambiguous grammars were found which were at least as effective as the best current unambiguous grammars.

**Conclusions:**

Overall the method of evolving SCFGs for RNA secondary structure prediction proved effective in finding many grammars that had strong predictive accuracy, as good or slightly better than those designed manually. Furthermore, several of the best grammars found were ambiguous, demonstrating that such grammars should not be disregarded.

## Background

RNA secondary structure prediction is the process of predicting the position of hydrogen bonds in an RNA molecule based only on its nucleotide sequence. These predictions can be used to better understand the functioning of cells, characteristics of gene expression and the mechanisms involved in protein production [1]. Early attempts at systematic prediction include [2]; who simply evaluated all possible structures with respect to free energy functions. Later, thermodynamic principles were used to advance free energy methods in algorithms such as UNAfold [3] and RNAfold [4]. (For a good overview of RNA secondary structure prediction, see [5]). Whilst energy minimisation models have proved popular, SCFG based methods also have their merits.

### Stochastic Context Free Grammars

A context–free grammar *G* (henceforth abbreviated to “grammar”) is a 4–tuple (N, V, P, S) consisting of a finite set *N* of non–terminal variables; a finite set *V * of terminal variables that is disjoint from N; a finite set *P* of production rules; and a distinguished symbol S ∈ N that is the start symbol. Each production rule replaces one non–terminal variable with a string of non–terminals and terminals.

One possible grammar, generating strings which may be interpreted as addition/multiplication expressions using only the number 1, may be represented thus:

(1) $$\begin{array}{lll} S & \to F+S|F\; & \;\\ F & \to 1|(S)|F*\mathrm{F.}\; & \; \end{array}$$

Note that each instance of *S* (standing for sum) generates a sum of n ≥ 1 terms (F+F+…F), and each *F* (standing for factor) generates a 1, a product of terms (F∗F), or a whole expression within parentheses. It should be clear that many different expressions might be generated: 1+1+1,(1+1∗1)+1+(1), and so forth. Formally, the grammar has non–terminal variables S, F, terminal variables (,),+,∗,1, production rules S→F+S,S→F,F→1,F→(S) and start symbol S. The order in which the production rules are used forms the *derivation* of a string. One valid derivation would be S⇒F⇒(S)⇒(F)⇒(1), generating the string ‘(1)’ and using the sequence of production rules S→F,F→(S),S→F,F→1.

A SCFG is a grammar with an associated probability distribution over the production rules which start from each T∈N. Beginning with the start symbol and following production rules sampled from the relevant distribution, a string of terminal variables can be produced (if the grammar terminates). Choosing nucleotide symbols or the three characters used in dot–parenthesis notation as terminal variables, SCFGs can be constructed which produce strings corresponding to nucleotide sequences or secondary structures.

### Application of SCFGs to RNA Secondary Structure Prediction

The use of SCFGs in RNA secondary structure prediction was based on the success of Hidden Markov Models (HMMs) in protein and gene modelling [6]. Any attempt to apply HMMs to RNA secondary structure was prevented by the long–range interactions in RNA [7]. SCFGs, being generalisations of HMMs, offer a solution. This was first exploited by [8] and then developed by others (e.g. [9]).

The Pfold algorithm [10,11] is one of the most successful approaches using SCFGs. It is designed to produce an evolutionary tree and secondary structure from an aligned set of RNA sequences. Pfold uses a SCFG designed specifically for RNA secondary structure prediction (denoted KH99 in this paper). Therefore, when only considering a single sequence, Pfold is simply a SCFG prediction method. There are other approaches which predict secondary structures from aligned RNA sequences, such as RNAalifold [12] and Turbofold [13]. However, we are concerned with the single–sequence prediction problem, so these are not used here.

While KH99 was effective, it seems to have been chosen relatively arbitrarily, in that there is little discussion about the motivation behind the choice of production rules. This problem was addressed by [14], in which nine different lightweight SCFGs were evaluated on a benchmark set of RNA secondary structures. The set of grammars they tested, however, was by no means exhaustive. All of these grammars were constructed by hand and there was little motivation for their production rules (except for the extension to stacking grammars: production rules such as $P^{b\hat{b}}\to aP^{\mathrm{aâ}}â$ for nucleotides $a,\;â,\;b,\;\hat{b}$). This suggested that a computational search of a large space of grammars might find stronger grammars, which is what we have attempted in this paper.

Evolutionary approaches have already been implemented for HMMs. Indeed, [15] used one to find the best HMM for protein secondary structure prediction. A mild improvement was found compared to HMMs which had been constructed by hand. It is hard to tell quite how conclusive the results were since the limited size of the data set forced training and testing to be done on the same data. Given the size of the data set, overtraining may have caused unreasonably high quality predictions. Clearly though, the method is potentially very powerful.

## Methods

In this paper, only *structure generating grammars* are considered (i.e. those which have terminal variables in {( , ), . }). Strings generated by these grammars uniquely define secondary structures, with a dot ‘.’ representing an unpaired nucleotide and an opening parenthesis indicating pairing with its corresponding closing parenthesis. Once a structure is generated, a generative model for sequences is to stochastically allocate nucleotides to each site according to the frequency of occurrence (paired and unpaired) in some set of trusted sequences and structures.

### Normal forms

To develop algorithms for analysing sequences under grammatical models, it is convenient to restrict the grammar to a normal form, with only a few possible types of productions. The normal form most commonly used is Chomsky Normal Form (CNF), as every context–free grammar is equivalent to one in CNF. However, a grammar in CNF cannot introduce the corresponding parentheses of paired nucleotides in a single production, and therefore does not capture structure in a straightforward manner. Thus it was necessary to create a new double emission normal form (so called because paired bases are emitted simultaneously) which was able to capture the fundamental features of RNA secondary structure: branching, unpaired bases, and paired bases. For any combination of non–terminals (*T, U, V* ) we allow only rules of the following form:

(2) $$\begin{array}{lll} T & \to \mathrm{UV}\; & \;\\ T & \to .\; & \;\\ T & \to (U)\; & \; \end{array}$$

This normal form allows the development of the structural motifs commonly found in RNA. For example (where V _i_ correspond to non–terminals) base–pair stacking can be generated by rules of the type $V_{1}\to (V_{1})$, hairpins by $V_{1}\to (V_{2}),\;V_{2}\to V_{2}V_{3}|V_{3}V_{3}$ and $V_{3}\to .\;\;$, and bulges by $V_{1}\to (V_{2})$, $V_{2}\to V_{3}V_{1}$ and $V_{3}\to .|V_{3}V_{3}\;\;$.

Furthermore, with the exception of the ability to generate empty strings, this normal form allows the expression of dependencies of any context–free grammar producing valid structures. It was also designed to avoid cyclical productions; that is, combinations of production rules which result in the same string that they started from. These are undesirable as they permit a countably infinite number of derivations for some strings. For this reason, rules of the form T→𝜖 and T→U were not considered. There is, however, nothing intrinsically wrong with these rules; it is quite possible to create strong grammars with these rules present (many of those used in [14] have rules of this type).

As a result of eliminating these rules, many grammars already established in RNA secondary structure prediction cannot be exactly replicated, since they are not initially in the above normal form. For example, the KH99 contains the rule S→L. As this normal form is an extension of CNF, any context–free grammar can be converted to this normal form, maintaining paired terminal symbol emissions. For example if S→L and L→.|(F), the forbidden rule S→L would be replaced by S→.|(F). This produces the same strings, and a given probability distribution for stochastic grammars can even be conserved. However, the transformation will often change the set of parameters in the model, which may result in different predictions when the production rule probabilities are inferred.

### Secondary structure prediction

Secondary structure can be predicted by two methods, both of which are employed here. Firstly, one can find the maximum likelihood derivation of a sequence, during which a structure is generated. The Cocke–Younger–Kasami (CYK) algorithm [16] determines, by dynamic programming, the probability of the most likely derivation, and so backtracking can be used to find the most likely structure. It is designed for grammars in CNF, though there are established methods of dealing with grammars in a different normal form [17].

Secondly, one can employ a posterior decoding method using base–pairing probability matrices. The base–pair probability matrix for a SCFG are obtained via the inside and outside algorithms [18]. The secondary structure with the maximum expected number of correct positions can then be calculated via dynamic programming. Our decoding algorithm follows [19], including a *γ* parameter specifying the trade–off between correct base pairs relative to correct unpaired positions. For assessing the fitness of a grammar in the genetic algorithm, a value of γ=2 was used, so as to maximise the expected number of positions correctly predicted.

Both methods were used in the search, as this additionally gave a chance to compare the two prediction methods.

### Ambiguity and completeness

A grammar is said to be *ambiguous* if it produces more than one derivation for a given structure [20]. If structure *A* has one derivation with probability 0.3, and structure *B* two derivations, each with probability 0.25, the CYK algorithm will choose structure A, while structure *B* is more probable. This may reduce the quality of predictions using the CYK algorithm. The posterior decoding, though, sums over all derivations in prediction, so might be affected less by grammar ambiguity.

Nine grammars are tested in [14], of which two are ambiguous. They find that the CYK algorithm does choose suboptimal structures, and that the ambiguous grammars perform poorly relative to the unambiguous grammars. Consequently, efforts have been made to avoid ambiguity [20]. The conclusion that ambiguous grammars are undesirable is not necessarily justified. The ambiguous grammars in [14] are small, with at most two non–terminal variables, and one might expect them to be ineffective regardless. Their poor predictive quality may be due to deficiency in design rather than ambiguity.

We define a grammar to be *complete* if it has a derivation for all possible structures which have no hairpins shorter than length two. Clearly the ability to generate all structures is desirable for a grammar. However, one should be careful not to overemphasise this desirability, even for a complete grammar, once parameters have been inferred, converting it to a SCFG, it is unlikely that all structures have a probability significantly different from zero for any sequence. Similarly, the posterior decoding is not prone to grammar incompletness in the same way that CYK is since, in theory, after obtaining the probabilities of unpaired and paired positions, structures which cannot be derived with the grammar can still be predicted. One can perform a heuristic test for completeness by testing on a sample of strings. Ambiguity can also be checked for heuristically [20], but determining grammar ambiguity and completeness is undecidable [21].

Practically, it is very difficult to ensure both unambiguity and completeness. A complete, unambiguous grammar cannot be simply modified without compromising one of the properties. Adding any production rules (if they are ever used) will create ambiguity by providing additional derivations. Equally, removing production rules will create incompleteness (unless the rule is never used in a derivation), as the original grammar is assumed unambiguous. Because of this, an automated grammar design based on simple–step modification is practically impossible without creating ambiguous and incomplete grammars. Moreover, grammars that are unambiguous and complete are vastly outnumbered by grammars that are not. Therefore, grammars not possessing these desirable qualities must be considered and as a result our grammar search serves as a test of the capabilities of ambiguous or incomplete grammars.

### Parameter inference

Training data, consisting of strings of nucleotides and trusted secondary structures, is used to obtain the probabilities associated with each production rule, as well as paired and unpaired nucleotide probabilities. If derivations are known for the training sequences, then there are simple multinomial maximum likelihood estimators for the probabilities. Usually, though, the derivation is unknown. Again, one can estimate probabilities by finding derivations for the training set using CYK, or by the inside and outside algorithms.

For the CYK algorithm, in the case of ambiguous grammars, one cannot know which derivation produced the known structure, so probabilities cannot be obtained. Consequently, we train these grammars using the same approach as [14] to ensure comparability. That is, we randomly select one derivation. For unambiguous grammars, such as KH99, this has no effect on the training. As with the prediction, inside–outside training works for unambiguous and ambiguous grammars alike.

Again, both CYK and inside–outside were used for parameter inference in the search and evaluation.

### Evolutionary algorithm

With the double emission normal form, for *m* non–terminal variables there are $2^{m^{3}+m^{2}+m}$ grammars ($m^{3}$ production rules of type T→UV, $m^{2}$ of type T→(U) and *m* of type T→.). An evolutionary algorithm would allow for efficient exploration of the space of grammars in the above normal form. The way that the algorithm searches the space is determined by the design of the initial population, mutation, breeding and selection procedure. To find effective grammars, these must be well designed.

#### Initial population

When forming the initial population, the size of the space of grammars quickly becomes problematic. The space is clearly large, even for small m, so the population size cannot approach that usually afforded in evolutionary algorithms [22]. We start with an initial population of small grammars, and use mutation and breeding rules which grow the number of non–terminal variables and production rules. Our initial population comprised sixteen grammars, of the form:

(3) $$\begin{array}{lll} S & \to \left(\left(\left\{\begin{array}{l} \mathrm{SS}\\ - \end{array}\right|\begin{array}{l} \mathrm{SB}\\ - \end{array}\left|\begin{array}{l} \mathrm{BS}\\ - \end{array}\right|\begin{array}{l} \mathrm{BB}\\ - \end{array}\right|\begin{array}{l} \left(S\right) \end{array}\right|\begin{array}{l} . \end{array}\; & \;\\ B & \to .\; & \; \end{array}$$

where between zero and four of the S→UV rules were excluded. We also tried initial populations containing the SCFGs from [14] to consider examining SCFGs close to these.

#### Mutation

Mutations constitute the majority of movement through the search space, so are particularly important. They give the grammar new characteristics, allow it more structural freedom, and add production rules which may be used immediately or may lie dormant. For non–terminals $V_{i}\in N$, and corresponding production rules $P_{V_{i}}$, the allowed stochastic mutations were: 

· The start variable (and corresponding production rules) change,

· A production rule is added or deleted,

· A new non–terminal variable $V^{\prime }$ is added along with two new rules that ensure that $V^{\prime }$ is reachable and that $P_{V^{\prime }}$ is not empty,

· A non–terminal variable is created with identical rules to a pre–existing one,

· A production rule of the form $V_{i}\to V_{j}V_{k}$ is changed to $V_{i}\to V_{j}V_{l}$, $V_{i}\to V_{l}V_{k}$ or $V_{i}\to V_{l}V_{p}$, or production rule of the form $V_{i}\to (V_{j})$ is changed to $V_{i}\to (V_{k})$.

This form of mutation is very basic, but allows many structural features to develop over generations. The rate of mutation determines movement speed through the search space and development of these structural features. Adding rules too slowly prevents grammars from developing structure, while too many results in a lot of ambiguity and thus creates ineffective grammars. Deleting rules almost always results in a worse grammar. To aid the grammar design, especially in consideration of facets of the normal form, the rule B→. was kept constant in the evolutionary process.

More complex mutation is clearly possible. The derivation could be used to find the rules used more often and make mutations of those rules more or less likely. A model for simultaneous mutations could be developed, which might be able to make use of expert understanding of RNA structure, in combination with an evolutionary search. We have found the above model to give sufficient mobility in the search space, and therefore did not investigate other extensions.

#### Breeding

The breeding model forms a grammar which can produce all derivations of its parent grammars. The grammar *G* formed from breeding *G*_1_and *G*_2_has start symbol *S*, non–terminals *V *_1_, *V *_2_, …, *V *_*n*_and *W *_1_, *W *_2_, …, *W *_*m*_, *B*, terminals .,(,) and production rules 

· $P_{S}=P_{S_{1}}\cup P_{S_{2}}$,

· For V_i_: $P_{V_{i}}$ where all occurrences of S_1_are replaced with S,

· For W_i_: $P_{W_{i}}$ where all occurrences of S_2_are replaced with S.

This breeding model was chosen to keep the size of the grammar relatively small, whilst allowing expression of both bred grammars to be present in derivations.

#### Selection

We grow the population in each generation by introducing a number of newly mutated or bred grammars, then we pare it back to a fixed population size by stochastic elimination. We determine the probability of elimination of a grammar by the inverse of some fitness measure. Fitness functions we use include mountain metric distances [23] between the predicted structure and the trusted structure, sensitivity (True Positives/(True Positives + False Negatives)) and positive predictive value (PPV) (True Positives/(True Positives + False Positives)). We follow the definition of accuracy in [14], where a base pair constitutes a true positive if it is present in both the predicted and true structures, false positive if present in only the predicted structure, and false negative if present in only the trusted structure. We also tried these accuracy measures in combination with other factors in the fitness function, including largest correctly predicted length, inability to predict structures, and cost for grammar complexity.

### Brute force search

In addition to the evolutionary algorithm, we have run a brute force search to evaluate small grammars which might be effective. One of the main points of emphasis of [14] was to look for *lightweight* grammars. In searching exhaustively for a small grammar, computational problems are quickly encountered. The search is feasible for grammars with only two non–terminals in addition to the rule B→. (16,384 grammars) but not for 3 non–terminals (over 500 billion grammars). Whilst the search in three non–terminals may be worthwhile, especially since KH99 contains only 3 non–terminals, it was far from computationally feasible for the purposes of this paper.

### Data

We took data from RNASTRAND [24], a collection of other databases [25-31]. We filtered the data set so that the sequences and structures could ensure reliability of predictions. We removed identical sequences and disregarded synthetic data and sequences with ambiguous base pairs. We further cleaned the data to filter out any sequences with greater than 80% base pair similarity with another structure (the standard used in [14]). Furthermore, we removed all sequences with pseudoknots as it is well established that SCFGs cannot predict pseudoknots [32].

The spectrum of sequence length, is of particular significance in selecting data. The CYK and training algorithms are of cubic order in the length of the string, so we decided to use large training and test sets with small strings. Longer strings require longer derivations, thus they have a larger weight in the parameter training, which might lead to overtraining. Equally, if one omits longer strings, poorer predictions may result from overtraining on the shorter strings. We found the trained parameters highly sensitive to the choice of training data set, and struggled to balance this with computational efficiency.

**Table 1:** Sensitivity and specificity of evolved SCFGs and other prediction methods

Grammar	$\mathrm{KH}99^{\prime }$	GG1	GG2	GG3	GG4	GG5	GG6	KH99	UNAfold	RNAfold	Pfold
Our data											
Sensitivity	0.496	0.505	0.408	0.413	0.474	0.469	**0.526**				
PPV	0.479	0.481	**0.551**	0.550	0.454	0.467	0.479				
F–score	0.478	0.441	0.473	0.470	0.461	0.339	**0.488**				
DE data											
Sensitivity	0.465	0.466	0.372	0.379	0.408	**0.487**	0.465	0.47	0.558	0.558	0.39
PPV	0.406	0.405	0.643	**0.646**	0.344	0.432	0.376	0.45	0.501	0.495	0.69
F–score	**0.480**	0.468	0.466	0.472	0.430	0.479	0.451				

The sensitivities, PPVs, and F–scores of grammars GG1–GG6 and $\mathrm{KH}99^{\prime }$ on the evaluation set and on the on the [14] data, as well as the benchmarked sensitivities and PPVs of UNAfoldv3.8 and RNAfoldv1.85 on the [14] data. Results for KH99 and Pfold are taken from [14].

We used a final data set from a variety of families, consisting of 369 sequences with corresponding structures. There was a total of 57,225 nucleotides with 12,126 base pairs. As with [14] and [10,11], sensitivity and PPV were used as the measures of structure fit, as well as F–score (2*True Positives/(2*True Positives + False Negatives + False Positives)), a more balanced measure of sensitivity and PPV. We investigated the practice of dividing data into training and test sets. [18] randomly generate training sets of 200 and 400 strings for parameter training. [14] use a training set of 278 structures and test set of 403 total structures. Meanwhile, [10] used a considerably larger training set, with 2273 structures, and a much smaller test set of just four sequences. Our training and test sets used in the evolutionary algorithm were disjoint sets of size 109, chosen at random from those sequences in our data of length less than 200 nucleotides. The remaining data form our evaluation set, on which grammars’ sensitivities and PPVs are reported.

As well as measuring performance on our own data, we have used results obtained with the [14] data. This helped us to verify the performances of the new grammars, to check our results against earlier work, and to verify that $\mathrm{KH}99^{\prime }$ is a good representation of KH99.

## Results and discussion

**Figure 1:**
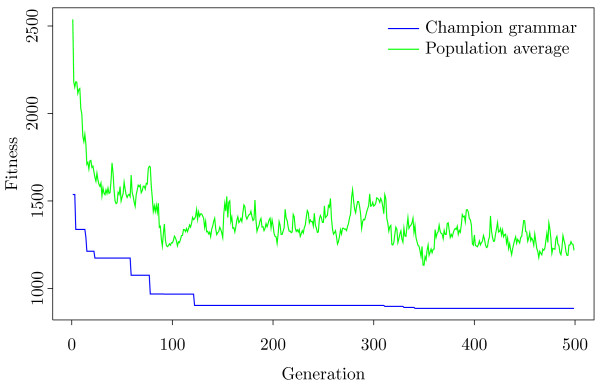
**Fitness evolution.** The change over generations in average fitness of population, and the fitness of the best SCFG. Here, a lower fitness is more desirable, the SCFG predicting better secondary structure. Many improvements to both the whole population and best SCFG are made in the first 100 or so generations. After this, the best SCFG does not become much better, but the average population fitness continues to fluctuate. Clearly the algorithm continues to explore alternative SCFGs and tries to escape the local optimum.

Figure 1 shows a typical realisation of the search process. The average fitness (here, smaller fitness is desirable) of the population is shown, as well as the fitness of the best recorded (champion) grammar. The average fitness of the population falls consistently as stronger grammars are found for approximately 100 generations, and then only minor improvements to the champion grammar were found. However, the population fitness continues to fluctuate as areas around the local optimum are searched.

Across all our experiments, over 300,000 grammars were searched. A number of strong grammars were found using both CYK and IO training and testing, denoted GG1–GG6. $\mathrm{KH}99^{\prime }$ is KH99 in the double emission normal form. Results on the sensitivity, PPV, and F–score of each grammar can be found in Table 1, in addition to the benchmark with the [14] data, and results on different training and testing methods can be found in Table 2. Table 2 also gives the scores of the combined best prediction, calculated by selecting, for each structure, the prediction with the highest F–score, and then recording the sensitivity, PPV, and F–score for that prediction.

(4) $$\begin{array}{lcr} A & \;\to & \mathrm{BA}|.|(C)\\ \mathrm{KH}99^{\prime }\;B & \;\to & .|(C)\\ C & \;\to & \mathrm{BA}|(C)\\ A & \;\to & \mathrm{AA}|\mathrm{BA}|.|(A)|(C)\\ \mathrm{GG}1\;B & \;\to & .|(C)\\ C & \;\to & \mathrm{BA}|(C)\\ A & \;\to & \mathrm{AA}|\mathrm{AB}|\mathrm{BA}|\mathrm{BB}|\mathrm{CB}|\mathrm{BC}|.|(B)|(C)\\ \mathrm{GG}2\;B & \;\to & .\\ C & \;\to & \;\;\mathrm{AA}|\mathrm{AB}|\mathrm{BA}|\mathrm{BB}|\mathrm{BC}|\mathrm{CA}|\mathrm{CB}|.|(A)|(B)|(\;C)\\ A & \;\to & \mathrm{AB}|\mathrm{BA}|\mathrm{BB}|\mathrm{AA}|\mathrm{DD}|(A)|(B)|(C)|(D)\\ \mathrm{GG}3\;B & \;\to & .\\ C & \;\to & \mathrm{AA}|.|(D)\\ D & \;\to & \mathrm{CD}|\mathrm{BD}|(A)|(C)\\ A & \;\to & \mathrm{CC}|\mathrm{CB}|\mathrm{BC}|\mathrm{EC}|(A)|(E)\\ B & \;\to & .\\ C & \;\to & \mathrm{CB}|\mathrm{BB}|(A)\\ \mathrm{GG}4\;D & \;\to & \mathrm{GC}|(C)\\ E & \;\to & \mathrm{AB}|\mathrm{CD}|.\\ F & \;\to & \mathrm{AB}\\ G & \;\to & \mathrm{FB} \end{array}$$

(5) $$\begin{array}{lcr} A & \to & \mathrm{DA}|\mathrm{CC}|.|(B)\\ B & \to & .\\ C & \to & \mathrm{AA}|\mathrm{HF}|(G)\\ \mathrm{GG}5\;D & \to & .|(E)\\ E & \to & \left(F\right)\\ F & \to & \mathrm{FB}|\mathrm{BF}|\mathrm{AA}|.|(A)|(F)\\ G & \to & \left(E\right)\\ H & \to & \mathrm{BG}\\ A & \to & \mathrm{DE}|\mathrm{AB}|\mathrm{BA}|\mathrm{AH}|.|(F)|(H)\\ B & \to & .\\ C & \to & \left(H\right)\\ \mathrm{GG}6\;D & \to & \mathrm{BB}|\mathrm{AC}\\ E & \to & .|(H)\\ F & \to & \mathrm{FB}|\mathrm{CF}|.\\ G & \to & \mathrm{GH}|(H)|(C)\\ H & \to & \mathrm{FA}|\mathrm{AF}|\mathrm{HH}|(B)|(H) \end{array}$$

**Table 2:** Sensitivity and specificity of evolved SCFGs using different training and testing methods

	Grammar	$\mathrm{KH}99^{\prime }$	GG1	GG2	GG3	GG4	GG5	GG6	Best
	Grammar found by		Local	IO	IO	CYK	CYK	CYK	
CYK	Sensitivity	0.496	0.505	0.330	0.374	0.474	0.469	**0.526**	0.675
	PPV	0.479	**0.481**	0.258	0.322	0.454	0.467	0.479	0.585
	F–score	**0.478**	0.441	0.426	0.435	0.461	0.339	0.461	0.622
IO	Sensitivity	0.387	0.392	0.408	**0.413**	0.373	0.404	0.410	0.450
	PPV	0.552	0.517	0.551	0.550	0.566	0.556	**0.583**	0.584
	F–score	0.461	0.443	0.473	0.470	0.449	0.471	**0.488**	0.493

The sensitivities, PPVs, and F–scores of grammars GG1–GG6 and $\mathrm{KH}99^{\prime }$ on the evaluation set, using different methods of training and testing. ‘CYK’ indicates that the CYK algorithm was used, and ‘IO’ that the inside and outside algorithms were used. The column ‘Best’ was calculated by selecting, for each structure, the prediction with the highest F–score, and then recording the sensitivity, PPV, and F–score for that prediction. It is perhaps not surprising that the ‘best’ predictions for CYK are better than the ‘best’ predictions for IO, as IO is in some sense averaging over all predictions. One might expect the predictions to be more similar than those from CYK, as seen by comparing IO values for GG6 and ‘best’, giving less increase when considering those with best F–score.

This shows grammars with very different structures perform well on the same (full evaluation) data set. $\mathrm{KH}99^{\prime }$ is still a strong performer, but we have shown that there exist many others which perform similarly (these GG1–GG6 form just a subset of the good grammars found in the search).

GG1 is $\mathrm{KH}99^{\prime }$ with two rules added, A→AA and A→(A). These rules were used infrequently (probabilities 0.007 and 0.047 respectively). The mild improvement in functionality allows for an additional fraction of base pairs to be correctly predicted.

GG2 and GG3 were found using the posterior decoding version of the evolutionary algorithm. They have a high density of rules, that is many rules for each non–terminal variable. Particularly, GG2 has almost all of the rules it is possible for it to have, given B→. is kept constant through the evolutionary algorithm. Given this, it is not surprising that they performed poorly using the CYK training and testing. However, with posterior decoding, the base–pair probabilities are still effective for good predictions.

GG4 has only two variables (A and C) used almost exclusively in producing base pairs. It then uses various exit sequences to generate different sets of unpaired nucleotides and returns to producing base pairs. Finally, GG5 and GG6 are typical of larger grammars we have found with complex structure. It is not obvious to us how their structure relates to their success in secondary structure prediction. GG4, GG5, and GG6 were all found using the CYK version of the evolutionary algorithm, and perhaps their complex structure can be accredited to this. GG6 is a strong performer throughout, particularly when considering F–score.

Most grammars achieved lower predictive power on the Dowell and Eddy dataset. The difference in performance between KH99 and $\mathrm{KH}99^{\prime }$ is small and confirms that the representation of KH99 as $\mathrm{KH}99^{\prime }$ is a good one. Particularly noteworthy is the performance of GG3 and GG5. GG3 has had a considerable increase in PPV, likely due to the posterior decoding prediction method. Given many of the structures in the Dowell and Eddy dataset contain pseudoknots, other grammars score poorly trying to predict pairs where there are not, in contrast to GG3. By predicting fewer base pairs, GG3 gains higher PPV as more of them are correct, but lower sensitivity. GG5 is a grammar which was unremarkable in its results on the original data set, however, it has outperformed the rest of the grammars on the benchmark set and is the only grammar with improved sensitivity when compared to the RNASTRAND dataset.

**Figure 2:**
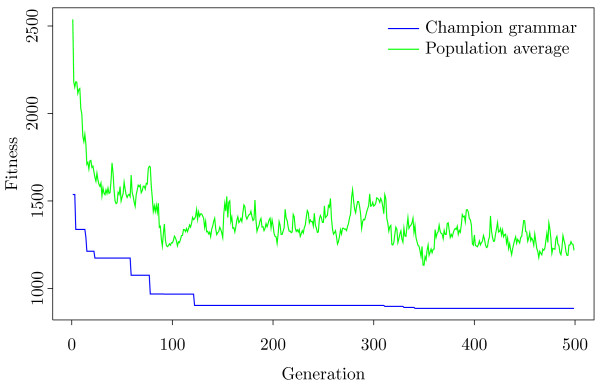
**Sensitivity/PPV curve.** A graph showing how sensitivity and PPV change for grammars when the posterior decoding parameter *γ* is changed to alter the frequency of base–pair prediction. The parameter *γ* ranged from 0.05 to 5 in increments of 0.05 for each grammar, and the sensitivity and PPV were measured on the full evaluation set. Grammar GG6 shows further strong performance here, having the largest area underneath the graph.

Figure 2 shows the sensitivity–PPV curve for grammars $\mathrm{KH}99^{\prime }$ and GG1–GG6. This was produced using the posterior decoding method by varying the parameter *γ*. GG6 again proves to do slightly better than the other grammars, having the largest area underneath the curve. $\mathrm{KH}99^{\prime }$ does not distinguish itself much from the other grammars, being in the middle in terms of area underneath the curve.

Overall, the grammars found in the evolutionary search still perform well because they are not overadapted to deal with the original data. Determining which is best depends on the measure of strength of prediction, whether the size of the grammar is a concern, ability to approximate structures with pseudoknots effectively, and so on. However, it is clear that a selection of effective grammars has been found. Results shown by UNAfold and RNAfold continue to be superior to those produced by SCFG methods.

**Figure 3:**
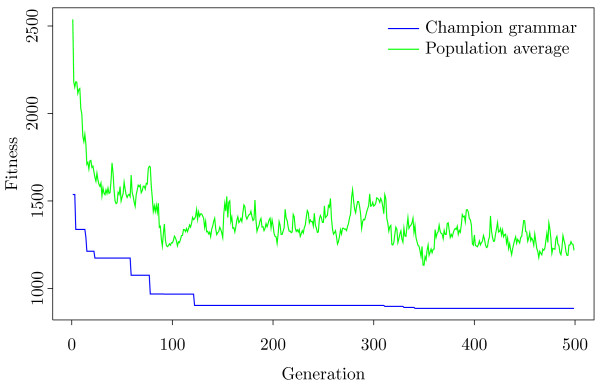
**Relative sensitivities/PPVs.** A heat map showing the relative sensitivities and PPVs of the different prediction methods, or between prediction method and known structure. $\mathrm{KH}99^{\prime }$ and GG1, produce very similar structures which is not surprising, given they were found by changing one and two rules of $\mathrm{KH}99^{\prime }$ respectively. Otherwise, the methods have relative sensitivities/PPVs of approximately 0.5−0.6, which is as expected, given they are all designed to predict RNA secondary structure. However, it is clear that they are markedly distinct from $\mathrm{KH}99^{\prime }$ in their structure predictions.

We also checked that the grammars obtained from the evolutionary algorithm do not merely produce similar structures to $\mathrm{KH}99^{\prime }$ by using different derivations. To do this we define the relative sensitivity of method A with respect to method B as the sensitivity of method A as a predictor of the structures produced by method B. The relative PPV is defined in a similar manner. We then compared the predictions of the grammars by building a heat map of the relative sensitivities and PPVs (Figure 3), using the evaluation set. As expected, $\mathrm{KH}99^{\prime }$ and GG1 predict almost identical structures, as they are highly similar. Similarly, it is perhaps not surprising that GG2 and GG3 have very similar predictions given they produce structure through posterior decoding. The rest of the methods have sensitivity and PPV relative to other prediction methods of approximately 0.4 – 0.6. As they are designed to predict RNA secondary structure using the same training set, one would expect some similarity in the predictions, although not as much as with $\mathrm{KH}99^{\prime }$ and GG1. This is confirmed by our results, suggesting that the new grammars produce different kinds of structures which are good representations of RNA secondary structure.

### Further analysis of 

To test the local features of the space, we evaluated variations of $\mathrm{KH}99^{\prime }$ against the full data set. Where a single rule was deleted, only one grammar had prediction accuracy of the order of $\mathrm{KH}99^{\prime }$. This is the grammar without the rule A→(C) (which would have been used only infrequently in $\mathrm{KH}99^{\prime }$, with probability 0.014). However, it is clear that deleting rules has a strong negative effect on the predictive power of $\mathrm{KH}99^{\prime }$, given that no others have sensitivity greater than 0.25. Of course, this might be expected given that this SCFG has been constructed manually, and it is therefore unlikely to have unnecessary production rules.

**Figure 4:**
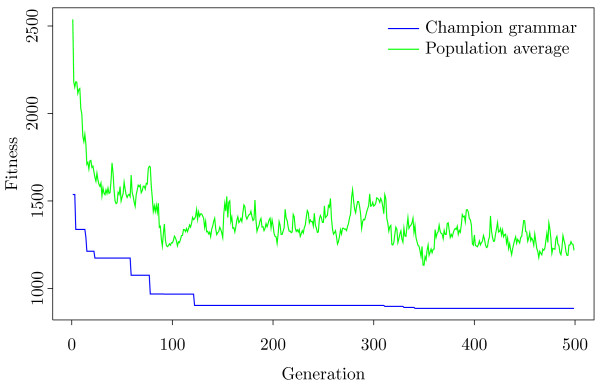
**Local search results.** Summary of the effects of adding one (giving 32 grammars) or two (giving 961 grammars) production rules to $\mathrm{KH}99^{\prime }$. The plot shows the cumulative proportion of grammars with given sensitivity. The grammars’ sensitivity is mostly still equal to the sensitivity of $\mathrm{KH}99^{\prime }$, with only a few outliers. GG1 was the top outlier for two production rule added. In this sense the space is reasonably flat.

With addition of rules, the number of grammars to check quickly becomes large. With one production rule added, 32 grammars must be evaluated, with two added this increases to 496. A similar local search for larger grammars would be impractical, since there are many more grammars with one or two altered production rules (for GG6, there are 584 grammars with only one new production rule, and 170,236 with two). Ambiguity of tested grammars had little or no effect. Results of this local search can be seen in Figure 4.

### Brute force search

**Figure 5:**
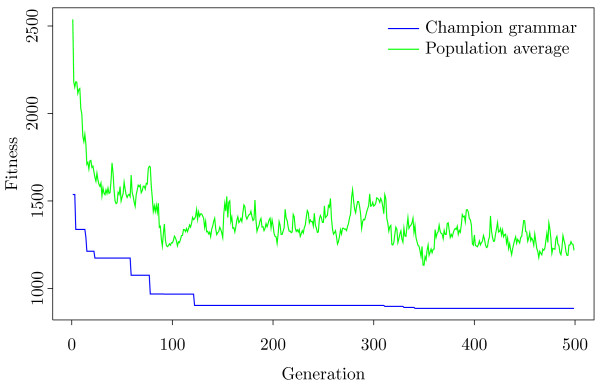
**Brute force search.** The distribution of sensitivity and corresponding PPV of grammars with at most 2 nonterminal variables. Approximately one quarter of grammars have sensitivity 0, as many cannot produce long strings. It is only the larger grammars that start to predict long strings which might correspond to structure. However, the prediction quality is still poor by both measures.

The brute force search illustrated how, with this normal form, larger grammars are needed to provide effective prediction. Most small grammars will only be able to produce one type of string. Also, it suggested that the existing grammars are close to locally optimal and that the space around them is quite flat, demonstrating the need for intelligent searching methods. Figure 5 illustrates the distribution of sensitivity across the space of grammars with at most 2 non–terminal variables. No grammar has sensitivity higher than 0.25 and approximately one quarter of grammars have sensitivity 0 (those which cannot produce long strings).

### Ambiguity and Completeness

One of the results of the search which we find most interesting is the ambiguity and completeness of GG1–GG6, shown in Table 3. All grammars found in the search were ambiguous, and still predicted structure effectively. In particular, ambiguous grammar GG1 performed better than $\mathrm{KH}99^{\prime }$, being a slight modification of it. Particularly, it is clear that GG2 and GG3 have many different derivations for each structure, and their strong performance relies on this ambiguity, as they perform poorly when tested with CYK. GG5 demonstrates further that ambiguous grammars can even be effective at approximating structures with pseudoknots. The effectiveness of some ambiguous grammars is likely due to the prediction algorithm picking structures that, whilst perhaps suboptimal, are close to what the best prediction would be. Clearly there is room for a further investigation into quite why some grammars cope better with ambiguity than others.

**Table 3:** Ambiguity and completeness

Grammar	Ambiguity	Completeness
$\mathrm{KH}99^{\prime }$	No	Yes
GG1	Yes	Yes
GG2	Yes	Yes
GG3	Yes	Yes
GG4	Yes	No
GG5	Yes	No
GG6	Yes	Yes

Ambiguity and completeness of $\mathrm{KH}99^{\prime }$ and GG1–GG6 grammars. All grammars found in the search were ambiguous. Some of the grammars found (GG4 and GG5), are incomplete but heuristically it seems that the structures that cannot be generated have little biological relevance.

Similarly, it might be surprising that some of the grammars found (GG4 and GG5), are incomplete. However, heuristically it seems that the structures that cannot be generated have little biological relevance (e.g. GG4 cannot generate “(…)(…)(…)(…)”). In some sense therefore, the incompleteness is permissible, as the grammar is still able to generate any relevant structure.

## Conclusions

Our brute force search and search around KH99 demonstrate that intelligent searching methods are necessary, and overall, the method of evolving SCFGs for RNA secondary structure prediction proved effective. We found many grammars with strong predictive accuracy, as good or better than those designed manually. Furthermore, several of the best grammars found were both ambiguous and incomplete, demonstrating that in grammar design such grammars should not be disregarded. One of the strengths of the method is the ease of application and effectiveness for RNA structure problems. In particular, grammatical models are used in phylogenetic models of RNA evolution [33] which make use of manually constructed grammars, and so the accuracy might be improved with automated grammar design. Overall though, whilst many grammars have been found with good predictive power, the space of grammars grows rapidly with the number of non–terminals, so we cannot conclude that no better grammars exist. The effectiveness of the search heuristic is supported by the fact that we consistently find grammars on par with the best manually created grammars. The equally consistent inability to achieve any significant improvement on this level of performance, and the relative limited prediction correlation between the many good grammars found points to the inherent challenge of grammar design, or indeed to the limitations of SCFG based methods as a whole. It appears that the number of grammars able to achieve this level of performance is large, and may depend little on the overall grammar structure, and at the same time it appears that a performance improvement may be difficult or impossible to achieve with a grammatical approach.

## Competing interests

The authors declare that they have no competing interests.

## Author’s contributions

JWJA conceived the idea in discussion with RL and JH. JWJA then developed the methodology with PT and JS, with help from RL. PT and JS then designed and wrote the code, and results were analysed and written up by JWJA, with help from PT and JS. All authors were involved in critical redrafting of the manuscript. All authors read and approved the final manuscript.

## References

[B1] Mayer C Neubert M Grummt I The structure of NoRC–associated RNA is crucial for targeting the chromatin remodelling complex NoRC to the nucleolus EMBO reports 2008 9 774 18600236 10.1038/embor.2008.109 PMC2515205

[B2] Pipas J McMahon J Method for predicting RNA secondary structure Proc Nat Aca Scien USA 1975 72 2017 10.1073/pnas.72.6.2017 PMC432683 1056009

[B3] Markham N Zuker M Keith JM UNAFold: software for nucleic acid folding and hybridization Bioinformatics, Volume II. Structure, Function and Applications 2008 Humana Press, Totowa 3 31 10.1007/978-1-60327-429-6_1 18712296

[B4] Hofacker I Fontana W Stadler P Bonhoeffer L Tacker M Schuster P Fast folding and comparison of RNA secondary structures Chem Mon 1994 125 167

[B5] Gardner P Giegerich R A comprehensive comparison of comparative RNA structure prediction approaches BMC Bioinf 2004 5 140 10.1186/1471-2105-5-140 PMC526219 15458580

[B6] Krogh A Brown M Mian I Sjölander K Haussler D Hidden Markov Models in computational biology: Applications to protein modeling J Mol Biol 1993 235 1501 8107089 10.1006/jmbi.1994.1104

[B7] Rabiner L A tutorial on Hidden Markov Models and selected applications in speech recognition Proceedings of the IEEE: February 1989 1989 257 286

[B8] Sakakibara Y Stochastic context–free grammars for tRNA modeling Nuc Acid Res 1994 22 5112 10.1093/nar/22.23.5112 PMC523785 7800507

[B9] Lefebvre F States DJ, Agarwal P, Gaasterland T, Hunter L, Smith RF A grammar–based unification of several alignment and folding algorithms Proceedings of the Fourth International Conference on Intelligent Systems for Molecular Biology 1996 AAAI Press, Menlo Park CA 143 154 8877514

[B10] Knudsen B Hein J RNA secondary structure prediction using stochastic context–free grammars and evolutionary history Bioinformatics 1999 15 456 10.1093/bioinformatics/15.6.446 10383470

[B11] Knudsen B Hein J Pfold: RNA secondary structure prediction using stochastic context–free grammars Nuc Acid Res 2003 31 3423 10.1093/nar/gkg614 PMC169020 12824339

[B12] Bernhart S Hofacker I Will S Gruber A Stadler P RNAalifold: improved consensus structure prediction for RNA alignments BMC Bioinf 2008 9 474 10.1186/1471-2105-9-474 PMC2621365 19014431

[B13] Harmanci A Sharma G Mathews D Turbofold: Iterative probabilistic estimation of secondary structures for multiple RNA sequences BMC Bioinf 2011 12 108 10.1186/1471-2105-12-108 PMC3120699 21507242

[B14] Dowell R Eddy S Evaluation of several lightweight stochastic context–free grammars for RNA secondary structure prediction BMC Bioinf 2004 5 71 10.1186/1471-2105-5-71 PMC442121 15180907

[B15] Won K Hamelryck TA Krogh A An evolving method for learning HMM structure: prediction of protein secondary structure BMC Bioinf 2007 8 357 10.1186/1471-2105-8-357 PMC2072961 17888163

[B16] Younger D Recognition and parsing of context–free languages in time n3 Inf Con 1967 10 189

[B17] Chappelier J Rajman M A generalized CYK algorithm for parsing stochastic CFG TAPD’98 Workshop 1998 Paris (France) 133 137

[B18] Lari K Young S The estimation of stochastic context–free grammars using the inside–outside algorithm Comput Speech Language 4 35

[B19] Choung D Wood D Batzoglou S CONTRAfold: RNA secondary structure prediction without physics–based models Bioinformatics 22 e90 16873527 10.1093/bioinformatics/btl246

[B20] Reeder J Steffen P Giegerich R Effective ambiguity checking in biosequence analysis BMC Bioinf 2005 6 153 10.1186/1471-2105-6-153 PMC1215473 15967024

[B21] Hopcroft J Motwani R Ullman J Introduction to Automata Theory, Languages and computation 2001 Addison Wesley, Reading, MA

[B22] Grefenstette J Optimization of control parameters for genetic algorithms IEEE Trans Syst Man Cybern SMC–16 1986 1 122

[B23] Moulton V Zuker M Steel M Pointon R Penny D Metrics on RNA secondary structures J Comp Biol 2000 7 277 10.1089/10665270050081522 10890402

[B24] Andronescu M Bereg V Hoos HH Condon A RNA STRAND: The RNA Secondary Structure and Statistical Analysis Database BMC Bioinf 2008 9 340 10.1186/1471-2105-9-340 PMC2536673 18700982

[B25] Andersen E The tmRDB and SRPDB resources Nuc Acid Res 2006 34 163 10.1093/nar/gkj142 PMC1347504 16381838

[B26] Berman H The nucleic acid database. A comprehensive relational database of three–dimensional structures of nucleic acids Biophys J 1992 63 3 751 1384741 10.1016/S0006-3495(92)81649-1 PMC1262208

[B27] Brown J The Ribonuclease P Database Nuc Acid Res 1999 27 314 10.1093/nar/27.1.314 PMC148169 9847214

[B28] Cannone J The comparative RNA web (CRW) site: an online database of comparative sequence and structure information for ribosomal, intron, and other RNAs BMC Bioinf 2002 3 2 10.1186/1471-2105-3-2 PMC65690 11869452

[B29] Griffiths-Jones S Rfam: annotating non–coding RNAs in complete genomes Nuc Acid Res 2005 33 121 10.1093/nar/gki081 PMC540035 15608160

[B30] Sprinzl M Vassilenko K Compilation of tRNA sequences and sequences of tRNA genes Nuc Acid Res 2005 33 139 10.1093/nar/gki012 PMC539966 15608164

[B31] Westbrook J Feng Z Chen L Yang H Berman H The Protein Data Bank and structural genomics Nuc Acid Res 2003 31 489 10.1093/nar/gkg068 PMC165515 12520059

[B32] Brown M Wilson C RNA pseudoknot modeling using intersections of stochastic context free grammars with applications to database search Pacific Symposium on Biocomputing 1996 1995 World Scientific Publishing Co., Singapore 9390227

[B33] Bradley R Pachter L Holmes I Specific alignment of structured RNA stochastic grammars and sequence annealing Bioinformatics 2008 24 23 2677 18796475 10.1093/bioinformatics/btn495 PMC2732270

